# Tracing the translator’s voice: A corpus-based study of six English translations of *Daxue*

**DOI:** 10.3389/fpsyg.2022.1069697

**Published:** 2023-01-12

**Authors:** Huiping Wang

**Affiliations:** Foreign Languages College, Shanghai Normal University, Shanghai, China

**Keywords:** translator’s voice, corpus-based study, English translation, *Daxue*, translation style

## Abstract

The Translator’s voice is the “second” voice in a translated narrative. By tracing a set of textual cues, the translator’s voice embedded in the discourse may come to light. This study is based on a self-constructed bilingual parallel corpus which adopts both quantitative and qualitative methods to reveal the translator’s voice in six English versions of the Chinese classic *Daxue*. WordSmith 8.0, CLAWS POS Tagger, and Readability Analyzer were used to analyze the data and investigate the lexical, syntactic, and textual features of the translations of *Daxue* by David Collie, James Legge, Ku Hungming, Ezra Pound, Chan Wing-Tsit, and Andrew Plaks, respectively. Paratexts of translation-prefaces, introductions, translator’s notes, footnotes, and so on, as well as representative examples, were also analyzed by close reading. The findings suggest that each translator’s cultural identity, historical background, and motives for producing the translation are made manifest through various linguistic and non-linguistic choices. This study demonstrates that the translator’s voice, which is always present along with the author’s voice and may take various forms, is deeply associated with the cultural and ideological constructs in which the act of translation is embedded. It is hoped that the results of this research will contribute to an enhanced understanding of the translation and overseas dissemination of the Chinese classic *Daxue*, and further the study of the translator’s voice with the aid of corpus technology.

## 1. Introduction

The Chinese classic *Daxue* (《大学》, *The Great Learning*) was originally a section of *Liji* (《礼记》 *The Book of Rites*), an anthology of treatises on rituals which came into shape around the first century BCE. After the edition and rearrangement by Zhu Xi (1130–1,200), the renowned Chinese scholar of the Song Dynasty, it gained its position as an independent “book” and was canonized as one of the so-called Four Books along with *Lunyu* (《论语》, *The Analects*), *Zhongyong* (《中庸》, *The Doctrine of the Mean*), and *Mengzi* (《孟子》, *Mencius*). Zhu Xi’s commentary edition of *Daxue* was one of the most important texts for education and the civil service examinations in China’s feudal society and had a profound influence on Chinese intellectuals for hundreds of years. Short as it is, the aim of *Daxue* is to produce an ideal political program based on an individual’s moral cultivation.

The Neo-Confucian of the Song Dynasty Cheng Yi (1033–1107) viewed *Daxue* as the entry point for the study of the Four Books: “*The Great Learning* (*Daxue*) is a surviving work of the Confucian school and is the gate through which the beginning student enters into virtue. It is only due to the preservation of this work that the order in which the ancients pursued their learning may be seen at this time” (Chan, 1963, p. 85–86). Its predominant position has made *Daxue* highly important and translated into various languages over the ages. So far, there have been approximately 18 important English versions of *Daxue*, as shown in Table 1.^1^

**Table 1:** Eighteen English translations of *Daxue* (including the translator’s name, book title, and publication year).

Translator(s)	Title	Year	Reference
Robert Morrison	*Ta-Hio: the Great Science*, included in *Horae Sinicae: Translations from the Popular Literature of the Chinese*	1812	Morrison, 1812
Joshua Marshman	*Ta-Hyoh*, included in *Elements of Chinese Grammar*	1814	Marshman, 1814
David Collie	*Ta Heo*, included in *The Chinese Classical Work Commonly Called The Four Books*	1828	-
James Legge	*The Great Learning*, included in *The Chinese Classics: with a Translation*, *Critical and Exegetical Notes*, *Prolegomena*, *and Copious Indexes* (*Vol*. *1*)	1861	-
Ku Hungming	*Higher Education*	1915	-
Lin Yutang	*Higher Education*, included in *The Wisdom of Confucius*	1938	Lin, 1938
Ezra Pound	*Ta Hio*, included in *The Great Learning of Confucius*	1928	-
	*Ta Hsio:The Great Digest*, included in *Confucius: The Great Digest & Unwobbling Pivot*	1947	-
E. R. Hughes	*The Great Learning*, included in *The Great Learning and the Mean-in-Action*	1942	Hughes, 1942
Chan Wing-tsit	*The Great Learning*, included in *A Source Book of Chinese Philosophy*	1963	-
Chai Chu	*Ta Hsueh* (*The Great Learning*), included in *The Sacred Book of Confucius*, *and Other Confucian Classics*	1965	Chai, 1965
A. Charles Muller	*The Great Learning*	1992	Muller, 1992
He Zuokang	*The Great Learning*, included in *The Great Learning & The Doctrine of the Mean*	1996	He, 1996
Irene Bloom	*The Great Learning* (*Daxue*), included in *Sources of Chinese Tradition: From Earliest Times to 1*,*600* (*2nd ed*.)	1999	De Bary and Bloom, 1999
Andrew Plaks	*Ta Hsüch* (*The Highest Order of Cultivation*), included in *Ta Hsüch and Chung Yung* (*The Highest Order of Cultivation and On the Practice of the Mean*)	2003	-
Ian Johnston/ Wang Ping	*Daxue* (*大学*)*: The Great Learning*, included in *Daxue and Zhongyong: Bilingual Edition*	2012	Johnston and Wang, 2012
Robert Eno	*The Great Learning*, included in *The Great Learning and the Doctrine of the Mean: Translation*, *Commentary and Notes*	2016	Eno, 2016
Wu Guozhen	*The Great Learning*, included in *Three Confucian Classics with Relevant Paraphrases*	2018	Wu, 2018

The English translation of *Daxue* has prompted investigations from various perspectives but they have primarily focused on one particular version. Wang (2005), for example, analyzed Pound’s etymographic interpretation in his translation. Wang and Ye (2008) suggested the socio-political setting of the time largely accounts for Morrison’s literal translation. Lu (2013) studied Legge’s version from the perspective of translation as adaptation and selection. Hou (2019) reviewed translation of *Daxue* and *Zhongyong* in philosophical terms of Hughes. There have also been several studies comparing and analyzing different versions of *Daxue*. For instance, Wang and Ye (2009) contrasted the translations by Morrison and Marshman. Fang (2011) compared Legge, Morrison, Marshman, and Lin’s versions from the perspective of functional translation theory. Chen (2020) studied the motives behind the English missionaries of the 19th century’s translations. Very little research uses the quantitative method to identify the features of the translated texts: Xu (2020), for example, examined the lexical features of the versions of six translators-Collie, Legge, Ku, Lin, Pound, and Muller, using text data mining, through R programming language. Despite the considerable insights gained through these studies on the translation of *Daxue*, there is remarkably insufficient research exploring the underlying mechanisms that produce the differences. Since the translation never coincides with its source and it always implies more than one voice in the text (Hermans, 1996), it is significant to find out how the translator’s individual voice is articulated in the translated text, what interventions exist, and to discover the underlying causes.

According to Chatman (1978, p. 153), voice “refers to the speech or other overt means through which events and existents are communicated to the audience.” Taivalkoski-Shilov (2015, p. 60) defines voice as “the set of textual cues characterizing a subjective or collective identity in a text.” As it marks the narrator’s identity, the concept of voice is central to narratology. Hermans (1996, p. 27) first gave the notion of the translator’s voice serious consideration, pointing out that translated narrative discourse always contains a “second” voice-the translator’s voice, which “may be more or less overtly present, and it is most directly and forcefully present when it breaks through the surface of the text speaking for itself, for example in a paratextual Translator’s Note.” O’Sullivan (2005, p. 109) claims that the discursive presence of the translator can be identified on two levels in the narrative text. The first is that of the implied translator as the originator of paratexts, and the second is the voice dislocated from that of the narrator of the source text. Munday (2008, p. 6) points out that “in translation studies, issues of style are related to the voice of the narrative and of the author/ translator.” He further elaborates on the relationship between voice and style as follows:

Whereas we shall use voice to refer to the abstract concept of authorial, narratorial, or translatorial presence, we consider style to be the linguistic manifestation of that presence in the text. Since the text is the only immediately visible part of the narrative, it is only by studying the language of the text that the style of the author or translator might really be identified and hence the voice(s) present in the discourse be determined. Voice is therefore to be approached through the analysis of style (Munday, 2008, p. 19).

In brief, the translator’s voice is a narratological term referring to the abstract concept of the translator’s discursive presence in a translated text, which “may be measured by creative linguistic choices as well as by repeated linguistic selections, both creative or standardizing, which relate to the idiolect of the translator and are manifestations of individual style” (Munday, 2008, p. 20).

According to Baker (2000), style is a kind of thumbprint expressed in a range of linguistic and non-linguistic features. To find out a translator’s style, it is therefore necessary to focus on the manner of expression that is typical of a translator and to capture their characteristic use of language in comparison with other translators, by drawing on corpus linguistics methods which can deal with a large body of data through the use of relevant software (Baker, 2000, p. 245–246). In recent years, corpus-related methodologies have been fruitfully applied to translation studies. Huang (2018) examined corpus-based studies of the translator’s style in China over the previous 2 decades and divided the research focus into two categories: *the comparative mode* focuses on the differences between a particular translator and other translators based on a collection of all or a number of their translated works; *the parallel mode* focuses on the translation of a single source text into a particular target language by various translators stressing the different approaches to handling specific aspects of the same original work (Huang, 2018, p. 79). These studies adopt literary works, primarily novels, plays, essays, and classical literature as their main sources, and are undertaken chiefly from three angles: descriptive translation studies, narratological stylistics, and computational linguistics (Huang, 2018, p. 80). Nevertheless, Hu and Xie (2017) pointed out the need to distinguish between “the translator’s style” and “the translation style” (or “the style of the translated text”). The former refers to a translator’s distinctive features that can be observed in a collection of all or a number of their translated works in comparison to those of other translators, manifested through their choice of translation materials, adoption of translation strategies or methods, language use in the translated text, and so on. The translation style refers to the distinctive features that distinguish one translated text from others while including elements of the styles of both the translator and the original work (Hu and Xie, 2017, p. 12–13). Accordingly, many current studies of the translator’s style (referred to as “the parallel mode” by Huang) are actually studies of the translation style.

It is noteworthy that several corpus-based studies on the translation style^2^ of ancient Chinese classics have been published concurrently. For instance, Zhao (2015) investigated two English versions of *Daodejing* (《道德经》, *Laozi’s Tao Te Ching*) from lexical, syntactic, and textual perspectives. Fan (2017) studied five English versions of *Lunyu* (《论语》, *The Analects*) by examining their linguistic features and translation strategies. Ge (2019) analyzed the macro-linguistic features of the four English versions of *Shangshu* (《尚书》, *The Book of History*). Previous studies have demonstrated the effectiveness of corpora as a tool for revealing the linguistic characteristics of various translated works. However, further investigation into the underlying causes of a variety of features of translation is still required. As Huang (2018) suggests, corpus studies of the translator’s (translation) style should be expanded to include semantic, pragmatic, and sociocultural analysis by incorporating ideas or methodologies from related fields, and by combining quantitative analysis and qualitative analysis to make the study more methodologically rigorous and scientific.

Since “all translating can be seen to have the translator’s subject position inscribed in it” (Hermans, 2014, p. 286), a translation’s style can be ultimately ascribed to the discursive presence of the translator in the text, that is, the translator’s voice. Munday (2008, p. 227) holds that style in translation is inherently non-systematic since none of the translators translates in the same way in all cases, yet there is always an element of choice and poetic taste. “The words we read in translation are the translator’s words, the result of translation choices” (Munday, 2009, p. 247); thus, the translator’s voice is how the translator establishes their identity as “the teller of the tale.” On these grounds, this study attempts to identify each translator’s voice in the six English translations of *Daxue* by examining the various translations’ styles using corpus methodology, together with a thorough qualitative analysis of the pertinent paratextual materials and representative examples of translation within the appropriate sociocultural, historical, and ideological framework. This study aims to provide a comprehensive and in-depth characterization of the six English versions of *Daxue* and to further the study of the translator’s voice with the aid of corpus technology.

Following this introduction, Section 2 will explain the materials and methods adopted in this study and why. Section 3 will present the results of the corpus analysis of the six English translations of *Daxue*. In Section 4, the implications of the results will be expanded upon, and the paratextual materials and illustrative examples will be further examined. Section 5 will offer the conclusions based on the research findings and highlight the limitations of this study with suggestions for the way forward.

## 2. Materials and methods

### 2.1. Materials

According to Baker (1995, p. 226), corpus refers to any collection of running texts, held in electronic form, and analyzable automatically or semi-automatically. A parallel corpus consists of an original text in language A and translated version(s) in language B. Parallel corpora contribute to the discipline of translation studies by supporting the emphasis shift from prescription to description (Baker, 1995, p. 230–231). This study is based on the parallel corpus of *Daxue*, in the Chinese original edited by Zhu (2011),^3^ and six English versions translated by Collie (1828), Legge (1861), Ku (2016),^4^ Pound (1947), Chan (1963), and Plaks (2003), respectively.

These translated texts were collected following the principles of representativeness and comparability. Firstly, the six translators are from different historical periods with divergent cultural identities, and their translations have had significant social and historical influence. According to Hall, there are two ways to conceptualize “cultural identity”: in terms of one, shared culture, which people with a shared history and ancestry hold in common, or as “what we have become,” taking into consideration the intervention of history (Hall, 1990, p. 223–225). Due to their individual socio-historical settings, the six translators each have a different cultural identity underpinning their individual motives and strategies of translation. Secondly, the source text of all six translations is one text-Zhu Xi’s commentary edition of *Daxue*. Prior to the corpora analysis, illustrative languages, such as prefaces, introductions, footnotes, translator’s notes, comments, and so on, were excluded from the analysis, to ensure the comparability of the materials for corpus analysis, producing the parallel corpus shown in Table 2.

**Table 2:** Description of the corpus in the current study.

Category	Tokens	Author/Translator
*Daxue*	2,830	Zeng Zi (edited by Zhu Xi)
*Ta Heo*	3,141	David Collie
*The Great Learning*	3,773	James Legge
*Higher Education*	3,694	Ku Hung-Ming
*Ta Hsio: The Great Digest*	4,265	Ezra Pound
*The Great Learning*	3,775	Chan Wing-Tsit
*Ta Hsüeh* (*The Highest Order of Cultivation*)	4,587	Andrew Plaks

### 2.2. Research tools

The corpus tools WordSmith 8.0, CLAWS POS Tagger, and Readability Analyzer were used in the study. WordSmith Tools are an integrated suite of programs developed by Mike Scott of Oxford University Press, including Wordlist, Keyword, and Concord programs which can look at how words behave in texts, produce a list of all the words or word clusters in a text, show any word or phrase in context, and help find the keywords in a text. CLAWS POS Tagger was developed by University Center for Computer Corpus Research on Language (UCREL) at Lancaster University, which offers a free tagging service online^5^ and generates output in either C5 or C7 tagset. Dr. Sarah Tyler developed the Readability Analyzer, which offers a free online readability analysis, passive voice detector, and other writing assistant services.^6^

### 2.3. Procedure

The analysis of the translated texts used WordSmith 8.0, CLAWS POS Tagger, and the Readability Analyzer. The corpora were processed by WordSmith 8.0, which automatically produced lexical and syntactic statistics. Once the translated texts had been tagged using CLAWS POS Tagger, the lexical density and conjunctions of each text were determined to reveal the lexical and textual features. The Readability Analyzer was used to determine the passivity and readability of the translated texts.

After the quantitative research was completed, an in-depth qualitative investigation of both the linguistic and non-linguistic differences between the six translations was carried out. The paratextual materials, such as prefaces, introductions, translator’s notes, footnotes, and so forth, were examined. Representative examples of the translated texts with a detailed analysis were given.

This combination of quantitative and qualitative approaches can map the textual traces of each translation thoroughly, reveal the translator’s discursive presence, or the translator’s voice, in each text, and provide a deeper understanding of these English translations of *Daxue*. Moreover, a further survey of the translator’s cultural identity and their motives for translating *Daxue* may reveal more about how their distinctive translators’ voices were formed and demonstrated in each English version of *Daxue* under investigation.

## 3. Results

### 3.1. Lexical features

The lexical features of the six translated texts (TT) were displayed using WordSmith Wordlist Program. The details include the number of tokens and types, type/token ratio (TTR, the ratio between type and token), standardized TTR (STTR is computed every 1,000 words as Wordlist goes through each text file), mean word length (MWL, the average length of words), and standard deviation (SD) of word length. Tokens are the number of words in each text, and types indicate the number of different words. TTR is a measure of the range and diversity of vocabulary used by a writer, or in a given corpus. Since the length of a text influences TTR, STTR is also provided by WordSmith, and considered more reliable (Baker, 2000, p. 250). MWL indicates the lexical complexity to some extent, and the standard deviation of MWL shows the length difference between words. The lexical statistics produced by WordSmith Wordlist are displayed in Table 3.

**Table 3:** Lexical statistics of the six TTs.

TT	Tokens	Types	TTR	STTR	MWL	SD
Collie	3,141	872	27.77	38.97	4.59	2.43
Legge	3,773	886	23.89	34.50	4.43	2.50
Ku	3,694	735	20.24	30.87	4.18	2.22
Pound	4,265	1,205	28.85	40.72	4.33	2.28
Chan	3,775	824	21.90	32.73	4.40	2.32
Plaks	4,587	1,079	23.55	36.00	4.58	2.54

As can be seen, Pound’s version has the highest STTR, suggesting that he adopts the most varied vocabulary in translation, whereas Ku’s version has the lowest STTR, indicating his translation uses the narrowest lexicon. Collie’s version has the longest MWL, closely followed by Plaks’ version, and Plaks’ SD is the highest of the six, implying that both Collie and Plaks tend to use long words, though Plaks’ vocabulary has greater variety. On the other hand, Ku’s MWL is the lowest of the six, closely followed by Pound, indicating relatively simple vocabulary used in their translations. The details of the word length of the six TTs are shown in Table 4.

**Table 4:** Word length details of the six TTs (in percentage).

TT	1-letter words	2-letter words	3-letter words	4-letter words	5-letter words	6-letter words	7-letter words	>8-letter words
Collie	2.32	17.64	23.43	14.87	9.93	10.88	6.84	14.09
Legge	3.18	20.75	22.98	15.05	9.94	9.01	5.67	13.42
Ku	4.03	18.84	23.31	19.06	12.83	8.01	5.09	8.83
Pound	3.77	17.89	23.56	17.14	11.21	9.12	7.34	9.97
Chan	2.25	19.36	22.91	16.95	11.39	9.40	5.77	11.97
Plaks	1.87	20.73	20.34	15.52	13.23	7.89	6.21	14.21

Ku’s version has the lowest percentage of words longer than eight letters, followed by Pound’s version, suggesting less lexical complexity in their versions. Plaks’ version has the highest percentage of words with more than eight letters, closely followed by Collie’s version, implying relatively complex word usage in their translation. This result is consistent with the previous findings in Table 3.

Moreover, the lexical density of the TTs was also calculated to illustrate their lexical features further. Lexical density (LD) is defined as the number of lexical words (or content words) divided by the total number of words. Lexical words are nouns, adjectives, verbs (excluding auxiliary verbs), and adverbs, which carry information about what is being communicated. LD is a measure of how informative a text is. The texts are tagged using CLAWS POS Tagger, and the statistics of the different parts of speech and LD are displayed in Table 5.

**Table 5:** Tagging statistics and LD of the six TTs (in percentage).

TT	Noun	Verb	Adj.	Adv.	LD
Collie	23.29	13.2	6.97	4.36	47.82
Legge	20.53	12.3	6.38	3.89	43.11
Ku	21.63	10.96	6.90	4.82	44.32
Pound	24.43	11.81	6.8	3.82	46.86
Chan	21.78	11.66	7.53	4.05	45.02
Plaks	22.80	11.10	8.59	5.08	47.57

It is evident that Collie’s version has the highest LD, closely followed by Plaks’ version, while Legge’s version has the lowest LD. This implies Collie and Plaks’ translations carry a greater information load, while Legge’s is the least informative of the six.

WordSmith Wordlist Program was then used to list the high-frequency words. A stop list is uploaded in advance to filter out irrelevant words (primarily function words), producing the top twenty high-frequency words of the six TTs as displayed in Table 6.

**Table 6:** Top 20 high-frequency words of the six TTs.

Order of Frequency	Collie	Legge	Ku	Pound	Chan	Plaks
1	VIRTUE	PEOPLE	PEOPLE	PEOPLE	PEOPLE	ONE’S
2	MAN	MAN	MAN	MAN	MAN	PEOPLE
3	PEOPLE	STATE	GOOD	HEART	STATE	MORAL
4	SAYS	BOOK	SAYS	STATE	ORDER	MAN
5	PRINCE	FAMILY	MEN	ORDER	WEALTH	CHARACTER
6	FAMILY	LET	ORDER	KING	MIND	MEN
7	FIRST	POETRY	NATION	OWN	MEANT	OWN
8	SHEWS	HIMSELF	MORAL	HAVING	BOOK	KINGDOM
9	ORDER	MEN	LOVE	ODES	ODES	WORLD
10	WHOLE	FIRST	MEANT	STRAIGHT	FAMILY	WEALTH
11	THINGS	VIRTUE	SONGS	SAY	SAYS	HUMAN
12	NATION	KNOWLEDGE	ORDERED	GREAT	RULER	WORDS
13	MIND	ABOVE	SAYING	HATE	GOOD	STATE
14	HENCE	THINGS	MIND	SEE	PERSONAL	CULTIVATION
15	OWN	WEALTH	BOOK	SELF	THEREFORE	SONGS
16	KNOWLEDGE	ABLE	ARIGHT	FAMILY	FIRST	PROPER
17	GOOD	GREAT	HOUSE	WORD	REMARK	MEANT
18	UPON	LOVE	WEALTH	ROOTED	SAYING	NOBLE
19	SUPERIOR	PERSON	FIRST	REST	LIFE	FIRST
20	MINISTER	EXPLAINS	CONDITION	MEN	KNOWLEDGE	FAMILY

It can be observed that the most frequently occurring words in four TTs are “people” and “man,” which is in accordance with the fundamental spirit of humanity of Confucianism, while Collie has “virtue” and Plaks has “one’s” as their most frequently occurring words, though “people” and “man” are frequent. In Pound’s version, “heart” occupies a prominent position, ranking 3rd in frequency. Ku and Plaks use the word “moral” more frequently than the other translators, ranking 8th and 3rd in frequency in their respective translations.

### 3.2. Syntactic features

WordSmith Wordlist Program was also used to calculate the number of sentences, the mean number of words in each sentence, and the standard deviation of the number of words between sentences, to reveal each translation’s syntactic features, as shown in Table 7.

**Table 7:** Syntactic statistics of the six TTs.

Syntactic Features	Collie	Legge	Ku	Pound	Chan	Plaks
Sentences	177	216	185	183	223	151
Mean (in words)	17.74	17.17	19.63	22.83	16.87	30.34
Std. Dev.	16.61	13.71	12.39	21.03	12.50	20.65

Of the six TTs, Chan’s has the most sentences with the shortest length and lowest standard deviation, suggesting a relatively simple syntactic structure. Following Chan closely, Legge has the second-shortest sentences and the second-highest amount of sentences. Plaks has the least number of sentences, but they have the most words, which suggests that Plaks tends to employ longer, more complex sentences. Pound uses the second-longest sentences overall yet with the highest standard deviation, suggesting that his syntactic structure is frequently intricate and not predictable.

The Readability Analyzer was adopted to determine the quantity and percentage of passive sentences. The results were then carefully checked to ensure accuracy. The statistics are in Table 8.

**Table 8:** Passive sentence statistics of the six TTs.

Passive Feature	Collie	Legge	Ku	Pound	Chan	Plaks
Passive sentences	29	73	28	36	55	62
Total sentences	177	216	185	183	223	151
Percentage	16.38	33.80	15.14	19.67	24.66	41.06

Plaks’ version uses more passive voice than the other five, followed by Legge’s TT, while Ku uses the fewest passive forms, followed by Pound’s TT. While active statements can sound more direct or personal, passive sentences typically sound more neutral and impersonal. The active voice might be preferable when giving oral presentations or in other more informal contexts (Wallwork, 2016, p. 66). As a result, compared to the four other translations, Plaks and Legge’s translations seem more impersonal and formal, while Ku and Pound’s translations seem more personal and casual, in form.

### 3.3. Textual features

One of the main differences between English and Chinese systems is that while English is characterized by hypotaxis (subordinate clause links through conjunctions, relative clauses, etc.), Chinese features parataxis. Parataxis refers to the connections between words or phrases being expressed through the logical relationships between them rather than through language use. This means that Chinese has far fewer conjunctions than English. When translating from Chinese into English, a translator needs to add conjunctions, for instance, so that English speakers can understand the text.

Baker (1996, p. 180) presents the notion of “explicitation” as one of the features of translated texts, which refers to “an overall tendency to spell things out rather than leave them implicit in translation.” However, explicitation may be norm-dependent, relating to a specific period of time or translation context, rather than universal (Olohan, 2004, p. 93). Schleiermacher argued that there are two approaches to translation: “Either the translator leaves the author in peace, as much as possible, and moves the reader toward him; or he leaves the reader in peace, as much as possible, and moves the author toward him” (Lefevere, 1977, p. 74). Venuti (1995, p. 20) referred to the former as the “foreignizing method”—an ethnodeviant pressure on those values to register the linguistic and cultural difference of the foreign text, sending the reader abroad, and the latter as the “domesticating method”—an ethnocentric reduction of the foreign text to target-language cultural values, bringing the author back home.

The use of conjunctions in translations from Chinese to English reflects the degree of explicitation and can reveal a translator’s propensity toward domestication or foreignization. To show the textual features of the TTs, CLAWS POS Tagger was used to calculate the usage of conjunctions in each text. The C5 tagset produced by CLAWS POS Tagger marks the conjunctions in three categories: CJC represents coordinating conjunctions, such as “and” and “or”; CJS represents subordinating conjunction, such as “although” and “when”; and CJT represents the conjunction “that.” The results are shown in Table 9.

**Table 9:** Statistics of conjunction usage of the six TTs (in percentage).

Textual features	Collie	Legge	Ku	Pound	Chan	Plaks
CJC	3.15	3.68	3.90	3.7	3.79	2.75
CJS	1.56	1.69	1.71	1.83	2.11	1.79
CJT	0.64	0.29	0.62	0.52	0.48	0.81
Total	5.35	5.66	6.23	6.05	6.38	5.35

Overall, Chan uses the highest percentage of conjunctions in translation, closely followed by Ku, while Collie and Plaks employ the least number of conjunctions of the six. This suggests that Chan and Ku are inclined to bridge the linguistic differences between English and Chinese in their translations to make the text more intelligible to the readers, suggesting a tendency toward domestication, while Collie and Plaks tend to maintain the textual features of the original, implying a tendency toward foreignization.

Moreover, to compare the overall readability of different TTs, the Readability Analyzer was applied once more, and the results (in the Flesch Reading Scale^7^) are shown in Table 10.

**Table 10:** Overall readability of the six TTs (in Flesch Reading Scale).

TT	Collie	Legge	Ku	Pound	Chan	Plaks
Overall Readability	60.22	65.40	75.49	71.30	67.44	58.16

Ku’s translation has the highest overall readability score, closely followed by Pound’s translation, indicating that both translations are fairly simple to understand. Plaks’ translation, on the other hand, is the least readable, closely followed by that of Collie, indicating that their translations are more challenging than the others.

## 4. Discussion

### 4.1. Translators’ voices as manifested by translation styles

As mentioned, to approach the nebulous translator’s voice requires first analyzing the translation style, that is, the linguistic manifestations of the translator’s voice in the translated text. The above results of the corpora analysis of the six English translations of *Daxue* reveal their respective linguistic features, and highlight the different translation styles, so enabling the translators’ voices from the textual traces left by their interventions to be mapped. Tracing the linguistic cues reveal that each translation has a distinctive style, which can be summed up succinctly in the following:

Collie’s translation has the longest mean word length and highest lexical density, with a relatively rich vocabulary (second-highest STTR), pointing to a complex lexicon and a significant amount of information. On the other hand, he uses fewer conjunctions than the other translators, making his translation relatively challenging to read (with the second-lowest readability score); Legge’s translation has the lowest level of lexical density, and the second-shortest mean sentence length as well as the second-highest percentage of passive sentences. This suggests that his translation style is concise and tends to be impersonal, with a moderate level of readability; Ku’s translation has the least varied vocabulary and the shortest mean word length (lowest STTR and MWL), making his lexicon appear to be straightforward and direct. His translation has the lowest percentage of passive sentences, presenting a personal and casual style. Ku’s translation also has the second-highest percentage of conjunctions and the highest readability; Pound’s translation has the richest vocabulary (the highest STTR) and the second-shortest mean word length, indicating a rich and straightforward vocabulary. His version also has the second-longest mean sentence length with the greatest standard deviation, pointing to a complicated and variable syntactic structure. His translation is generally rather straightforward to understand (with the second-highest readability score); Chan’s translation has the most sentences and the shortest sentence length, indicating a simple syntactic structure. On the other hand, his version contains the highest proportion of conjunctions, implying his translation tends to bridge the textual differences between English and Chinese to make the English translation comprehensible to the target readers, so making his translated text relatively readable; Plaks’ translation has the second-longest mean word length, with the highest standard deviation, and the highest percentage of words longer than eight letters, indicating a challenging and varied vocabulary. Plaks’ translation also has the second-highest lexical density, indicating a sizeable information load. His version is the most challenging to read, with the longest mean sentence length of the six, the highest frequency of passive sentences, and the lowest percentage of conjunctions (with the lowest readability score).

Example (1) below, extracted from the six translations, further illustrates the different styles of the translations:

1. ST: 大学之道, 在明明德, 在亲民, 在止于至善。

Collie: Superior learning consists in clearly illustrating brilliant virtue, renovating the people, and resting only in the summit of excellence.

Legge: What the Great Learning teaches, is to illustrate illustrious virtue; to renovate the people; and to rest in the highest excellence.

Ku: The object of a Higher Education is to bring out(明)the intelligent(明)moral power(德)of our nature; to make a new and better society (lit. people); and to enable us to abide in the highest excellence.

Pound: The great learning [adult study, grinding the corn in the head’s mortar to fit it for use] takes root in clarifying the way wherein the intelligence increases through the process of looking straight into one’s own heart and acting on the results; it is rooted in watching with affection the way people grow; it is rooted in coming to rest, being at ease in perfect equity.

Chan: The Way of learning to be great (or adult education) consists in manifesting the clear character, loving the people, and abiding (*chih*) in the highest good.

Plaks: The Way of self-cultivation, at its highest level, is a three-fold path:

it lies in causing the light of one’s inner moral force to shine forth,

in bringing the people to a state of renewal,

and in coming to rest in the fullest attainment of the good.

Example (1) is the opening line of *Daxue*, which is the central theme of the entire book, expounding the ultimate goal of *Daxue*- the “Three Items”(三纲 *san gang*), or more specifically, “明明德” (*ming ming de*, literally, to brighten up the bright virtue. The first “明” is a verb, meaning “to brighten up”, and the second is an adjective, meaning “bright”), “亲民” (*qin min*, literally, to love the people. According to Zhu Xi’s annotation, “亲民” is the same as “新民,” *xin min*, literally, to make the people new) and “止于至善” (*zhi yu zhi shan*, literally, to stop at the utmost goodness).

Collie mostly keeps the original meaning but adds two adverbs “clearly” and “only” to strengthen the tone. In comparison with the other versions, his translation employs a more formal vocabulary, such as the use of “superior learning” instead of “great learning” to translate “大学” (*daxue*).

Legge translates “明明德” as “to illustrate illustrious virtue,” using two English words with the same root to conform to the two “明” in the original. His syntactic structure mimics the original through three to-infinitives to correspond to the three “在” (*zai*, literally, to lie in) in Chinese. His usage of short sentences maintains the brevity of the original.

Ku’s translation adopts a simpler lexicon (such as the use of the verbal phrase “bring out” to translate the first “明”), and he heavily emphasizes his interpretation by rendering “德” as “moral power of our nature,” and “亲民” as “make a new and better society.” He keeps the original Chinese characters of the keywords to fully convey the meaning and his use of the first-person pronouns “our” and “us” exhibits a highly personal style. Overall, his translation is quite simple to read.

Pound adopts a unique etymographic interpretation whereby the meaning of words comes from ideograms. For instance, he broke “德” into several components including “彳” (a small step or pace), “罒” (eye), and “心” (heart), and then produced his own interpretation of this character as “the process of looking straight into one’s own heart and acting on the results.” Moreover, the Chinese character “親” (“亲”) is separated by Pound as “亲” (affection) and “见” (to watch), so that “亲民” becomes “watching with affection the way people grow.” Pound’s unique etymographic interpretation accounts for some of his lengthy sentences and the rich vocabulary of his version. The word “heart” has an abnormally high frequency in Pound’s version due to the fact that quite a few Chinese characters contain the component “心” (heart).

Chan’s translation demonstrates his profound understanding of the key concepts of the original. For example, “大学” is translated as “The Way of learning to be great (or adult education),” which accurately captures the nuanced meaning of the original. In addition, he translates “亲民” as “loving the people,” which he believes is more in accordance with the fundamental Confucian spirit of “humanity.” He also utilizes transliteration in addition to his translation of “止”— “abiding (*chih*) in” to explain the intricate meaning of the Chinese character. Chan adopts short sentences, and his translation is relatively easy to understand.

Plaks uses more challenging vocabulary, and his translation includes a wealth of additional information based on his in-depth understanding of the original. For example, “大学之道” (*daxue zhi dao*, literally, the way of adult learning) is translated as “The Way of self-cultivation, at its highest level, is a three-fold path,” with words added to the translation to elaborate on the inferred meaning for the target readers. His translation attempts to convey the complicated connotations of the original by delving into great detail, resulting in over-wordy sentences and rendering the translated text relatively difficult to read.

### 4.2. Reasons: Translators’ different cultural identities and motivation

Baker (2000, p. 258) points out that identifying linguistic habits and stylistic patterns is not an end in itself: it is only worthwhile if it tells us something about the cultural and ideological positioning of the translator, or about the cognitive processes and mechanisms that contribute to shaping their translational behavior. Therefore, it is necessary for us to ponder over the potential motivation for the stylistic patterns discovered from the corpus analysis.

The six translators under investigation have different cultural identities as well as different motivations for translation, which prompts them to translate in diverse manners. Baker (2000) believes style in translation involves the translator’s choice of the type of material to translate, his or her consistent use of specific strategies-including the use of prefaces or afterwords, footnotes, glossing in the body of the text, and so on. Hermans (1996) claims that the translator’s voice is most directly and forcefully present when it breaks through the surface of the text speaking for itself, for example, in a paratextual Translator’s Note. As the translator’s voice may be more overtly present in paratexts,^8^ an analysis of paratextual materials can further reveal the translator’s discursive presence. The next section investigates the paratexts of translation (prefaces, introductions, translator’s notes, footnotes, and so on) and elaborates on the translators’ cultural identities and translation motives within their specific sociocultural, historical, and ideological contexts by analyzing representative examples, allowing the translators’ voices in the different versions of *Daxue* to emerge fully and the factors that led to their formation to be clarified.

#### 4.2.1. The 19th-century missionaries: To evangelize

Both David Collie (?—1828) and James Legge (1815—1897) were missionaries dispatched by the London Missionary Society in the 19th century. Collie, however, was never allowed to visit China due to the policy which banned the entry of foreigners into China in the early 19th century. While serving as the president of the Anglo-Chinese College in Malacca, Collie completed the translation of the Four Books, containing the English translation of *Daxue*. He stated his translation purpose clearly in the preface:

The following Version of the Four Books was undertaken, in the first instance, for the purpose of acquiring some knowledge of the Chinese language…It might perhaps be of some use to the Chinese who study English in the College, not only by assisting them in acquiring the English Language, but especially in leading them to reflect seriously on some of the fatal errors propagated by their most celebrated sages(Collie, 1828: p. i).

It is clear that Collie’s translation serves two main functions: to serve as a tool to learn Chinese and to prove that Confucianism has fundamental errors in both religion and morality. To highlight the exotic nature of this Confucian classic, Collie generally adopted the foreignizing approach, which resulted in the complex lexicon, scarcity of conjunctions, and poor readability level of his translation. He did, however, occasionally omit or deviate from the original, inserting his own interpretation. Example (2) below is extracted from the translations by Collie and Legge:

2. ST: 古之欲明明德于天下者, 先治其国； 欲治其国者, 先齐其家； 欲齐其家者, 先修其身； 欲修其身者, 先正其心； 欲正其心者, 先诚其意； 欲诚其意者, 先致其知, 致知在格物。

Collie: The ancient (Princes) who felt desirous that the brilliancy of resplendent virtue might shine through the whole Empire, first promoted good order in their own provinces;—wishing to establish order in their own provinces, they first regulated their own families;—in order to effect the regulation of their own families, they first adorned their persons with virtue, in order that they might adorn their persons with virtue, they first rectified their own hearts; wishing to rectify their hearts, they first purified their motives; in order to purify their motives, they first extended their knowledge to the utmost.

Legge: The ancients who wished to illustrate illustrious virtue throughout the empire, first ordered well their own States. Wishing to order well their States, they first regulated their families. Wishing to regulate their families, they first cultivated their persons. Wishing to cultivate their persons, they first rectified their hearts. Wishing to rectify their hearts, they first sought to be sincere in their thoughts. Wishing to be sincere in their thoughts, they first extended to the utmost their knowledge. Such extension of knowledge lay in the investigation of things.

Example (2) involves the essential “Eight Steps” (八目, *ba mu*)of *Daxue*, which are the blueprints for translating humanity into actual living (Chan, 1963, p. 84). Collie omitted the last part of this passage-“致知在格物” (*zhi zhi zai ge wu*), which is also regarded as the most fundamental, while Legge translated it faithfully as “Such extension of knowledge lay in the investigation of things.” Besides, Collie’s translation also features a significant number of lengthy footnotes, including both “the substance of various Comments” and “the remarks of the Translator,” the latter reflecting Collie’s derogatory attitude toward Confucianism. In a footnote on the first page of his translation of *Daxue*, he refuted the Confucian thought by claiming:

The above passage looks beautiful in theory, and contains some important truths, but there is one grand fallacy at the foundation of the system…it is only the right knowledge of the Creator, and Savior of the world and a spiritual renovation by the power of the Divine Spirit, that can produce that purity of heart, singleness of intention and moral rectitude of conduct (Collie, 1828, p. 1).

It is evident that the omission by Collie was deliberate due to his rejection of the system of Confucian thought. Collie, however, tended to amplify and interject his own understanding into the translation of several other passages. For example, “修其身” was translated by Collie as “adorn their persons with virtue,” while Legge rendered it more literally into “cultivate their persons.” According to Collie, “virtue,” the most frequently used word in his translation, was endowed by, and could only be replenished by God: “It is to be feared that the standard of perfect virtue, formed by the Chinese Philosophers is very low, hence the ruinous notion that man may, unaided by divine influence, make himself perfectly virtuous…” (Collie, 1828, p. 3) Hence, “修身” (literally, cultivate oneself) was repeatedly rendered into “adorn one’s persons with virtue” by Collie, hinting that individuals need to resort to divine assistance to make themselves virtuous. Collie’s added interpretations also increased the information load of the translated text, leading to high lexical density.

In response to criticism of his translation by some claiming that “in many instances the rendering is too literal and in others too free, in many cases the spirit and force of the original have been lost,” Collie defended himself in the preface:

In fact, when he (the Translator) considers the comparatively little value of the work, and the important engagements which form his proper employment, he feels that he ought rather to apologize for having bestowed so much time upon it, than for not having succeeded in giving a good and faithful version (Collie, 1828: p. vi).

According to Collie, translating *Daxue* was a work of “comparatively little value,” and his “important engagements” were to preach Christianity to the Infidels who believe in Confucianism; therefore, faithfulness to the original was not the topmost priority for him.

More fortunate than Collie, Legge managed to arrive in Hong Kong as head of the Anglo-Chinese College when it was moved from Malacca to Hong Kong in 1843, and it was during his stay there that he gained a deep understanding of Chinese culture. He believed that studying the Confucian classics was crucial for foreign missionaries to better appreciate Chinese culture and philosophy to facilitate their missionary work in China. In the preface of *The Chinese Classics* (*Vol*. *1*) he states that he undertook the translation of Chinese classics with the following conviction:

…that he should not be able to consider himself qualified for the duties of his position (as a missionary), until he had thoroughly mastered the Classical Books of the Chinese, and had investigated for himself the whole field of thought through which the sages of China had ranged, and in which were to be found the foundations of the moral, social, and political life of the people (Legge, 1861, p. vii).

Consequently, Legge was determined to study Chinese culture and translate the Confucian classics, gradually turning himself into a sinologist. Although Legge had read the various views of scholars extensively to help him understand the classics, his translation objective “has been to condense rather than expand” (Legge, 1861, p. x). Since his missionary colleagues were primarily his target readers, he adopted a simple and flexible vocabulary to create a smooth and accessible translation while also taking the needs of students and general readers into consideration: “He (the Translator) hopes that the volumes will be of real service to Missionaries and other students of the Chinese language and literature. They have been foremost in his mind as those whom he wished to benefit. But he has thought also of the general reader” (Legge, 1861, p. ix). Thus, Legge’s translation has low lexical density, short sentences, and a medium degree of readability. *The Chinese Classics* contains long prolegomena, in which Legge went to great lengths to discuss the history, arrangement, authorship, scope, value, and so on, of *Daxue*. Overall, Legge was highly reverent of this Chinese classic:

First, the writer conceives nobly of the object of government, that it is to make its subjects happy and good…Second, The insisting on personal excellence in all who have authority in the family, the State, and the empire, is a great moral and social principle…The work which contains those principles cannot be thought meanly of (Legge, 1861, p. 33-34).

It was precisely out of his esteem for this Chinese classic that Legge had a more objective viewpoint than Collie and attempted to accurately communicate the original meaning without undue alteration. He frequently used passive forms to convey an impersonal (more direct and honest) tone. Meanwhile, Legge also employed a great many lengthy footnotes to further express his understanding of the work, but in contrast to Collie, Legge was mainly concerned with demystifying linguistic and conceptual problems, in order to help readers appreciate the original better. Legge went to great lengths to provide detailed and comprehensive annotations on almost every section of the original. As a result, his translation has been widely recognized as the authoritative one.

#### 4.2.2. Patriots in times of social upheavals: To salvage

The Chinese scholar Ku Hungming (1857—1928) was born in Malaysia to a Chinese immigrant family. He acquired his early education abroad and became proficient in western languages and culture. He fell in love with traditional Chinese culture after returning to China. After the Sino-Japanese War, a number of insightful intellectuals began to enthusiastically learn from the west in an effort to save the nation. This led to a trend to translate and transfer western knowledge into Chinese at the expense of ignoring China’s own culture and thoughts. Ku set himself against this tide by promoting traditional Chinese culture to the west by writing books and translating the Chinese classics, which he believed was the real remedy to save the country.

Despite the credit given to Legge’s translation as the orthodox version, Ku maintained that Legge’s translation lacked a thorough understanding of ancient Chinese philosophy and that his language was awkward. In the preface to his translation of *Zhongyong*— *The Conduct of Life or the Universal Order of Confucius*, Ku claimed:

My object, after I have thoroughly mastered the meaning, is not only to reproduce the matter, but also the manner of the original… But to be able to reproduce the manner— what in literature is called the style— of the great and wise men of the past, one must try to put oneself in the same state of mind as that to which they attained… (Ku, 1920, p. 9)

To familiarize modern western readers with ancient Chinese thoughts, Ku adopted domesticating approach in translation, replacing Chinese culture with western cultural components which the target readers were more familiar with. The translations of Ku and Legge can be compared in Example (3) below:

3. ST: 富润屋, 德润身, 心广体胖, 故君子必诚其意。

Ku: Wealth embellishes a house, but moral qualities embellish the person. When the mind is free and easy, the body will grow in flesh. Therefore a gentleman must have true ideas.

(*In order to have true ideas*, *Matthew Arnold says you must see “the object as in itself it really is*,*” and in order to do that*, *“you must get yourself out of the way*.*”*)

Legge: Riches adorn a house, and virtue adorns the person. The mind is expanded, and the body is at ease. Therefore, the superior man must make his thoughts sincere.

In Example (3), “诚其意”(*cheng qi yi*) in the original sentence was translated literally by Legge as “make his thoughts sincere,” while Ku rendered it as “have true ideas,” immediately followed by a note in the brackets quoting a statement by the English poet and literary critic Matthew Arnold to explain what it means to “have true ideas.” With such cultural transplantation, Ku endeavored to eliminate the exoticism of Chinese culture for western readers, providing them with a sense of cultural consensus. Moreover, Ku translated “德” as “moral qualities,” for which Ku gave this reason:

…the Chinese civilisation is a moral and true civilisation because in the first place it not only recognizes this moral obligation as the fundamental basis of its social order, but it makes the perfect attainment thereof in men its sole aim…In the following translation then this idea of moral obligation, which forms the basis of human conduct and social order in the scheme of the Chinese civilisation, will be explicitly set forth (Ku, 1920, p. 13).

Ku’s emphasis on the idea of moral obligation in his translation accounts for the high frequency of the word “moral.”^9^ Ku employed straightforward vocabulary, many conjunctions, and a casual and personal tone to make the translation more accessible to westerners unfamiliar with Confucian thought, making his translation highly readable. His domestication strategy to ingratiate himself with western readers was a compromise in light of the specific historical context. Yet it is undeniable that Ku’s translation broke down cultural barriers and promoted the transfer of Chinese culture and philosophy to the west to a certain extent.

Ezra Pound (1885–1972) also lived through a period of upheaval, much like Ku. Pound is mostly recognized as an American poet in the 20th century. Actually, he was also quite an accomplished translator, inseparable from his identity as a poet. “Pound’s poetry is translation and his translation is innovative poetry,” claimed Alexander (1979, p. 18). In the first half of the 20th century, the Western world was undergoing a series of crises, ranging from social, political, and economic to spiritual beliefs. The emphasis on self-cultivation and social order of Confucian thoughts aroused interest of Pound (1928). In 1915 Pound published *Cathay*, an anthology of Chinese classical poems, after he had studied Fenollosa’s notebooks. He then developed an interest in Chinese characters. The first Confucian text he translated was *Ta Hio*, *The Great Learning of Confucius* in 1928, which was based on Pauthier’s French version of the Four Books. After teaching himself Chinese *via* dictionaries, Pound was able to produce *The Great Digest* based on the original Chinese work, first in Italian in 1942 and then in English in 1947.

To Eliot’s question, “What does Mr. Pound believe?” Pound answered explicitly, “I believe the *Ta Hio* (*Daxue*)” (Zhu, 2005, p. 57). In the preface to *The Great Digest*, Pound remarked:

China was tranquil when her rulers understood these few pages (of *Daxue*). When the principles here defined were neglected, dynasties waned and chaos ensued. The proponents of a world order will neglect at their peril the study of the only process that has repeatedly proved its efficiency as social coordinate (Pound, 1947, p. 19).

Pound believed Confucianism was an “immediate need” to save western civilization. The main theme of *Daxue* is the significance of self-cultivation that extends from the regulation of a family, the well-ordered state, and finally, to a tranquil and happy world, which Pound believed would provide a “blueprint for world order in the future” (Cheadle, 1997, p. 2).

Lan (2005, p. 27–29) points out that central to Pound’s interpretation of Confucius is his unique approach to Chinese characters, referred to as “etymographic reading.” This approach results partly from the influence of Fenollosa’s study of the ideogram on Pound, and partly from Pound’s own determination to look for expressions that could differentiate his translations from those of his predecessors. Pound believed that the translator should treat a Chinese character as a palimpsest-that is, recognize the multiple-layeredness of the word, and aim to penetrate to the core of its meaning (Lan, 2005, p. 32). Example (4) is extracted from the translations by Pound and Legge:

4. ST: 《诗》 云: “緍蛮黄鸟, 止于丘隅。”

Pound: The *Book of Poems* says:

 The twittering yellow bird,

 The bright silky warbler.

 Talkative as a cricket.

 Comes to rest in the hollow corner.

 of the hill.

 

Legge: In the *Book of Poetry*, it is said, “The twittering yellow bird rests on a corner of the mound.”

Example (4) is a line of poetry from *Shijing* (《诗经》, the *Book of Poems*) “緍蛮黄鸟, 止于丘隅” (*min man huang niao*, *zhi yu qiu yu*). Legge translated it faithfully as: “The twittering yellow bird rests on a corner of the mound.” In comparison, Pound’s version appears more in poem form, with the translated text in five lines. Pound creatively translated “緍蛮” (literally, the sound of a bird) in terms of etymographic interpretation. To him, these two characters are composed of “日” (bright), “纟” (silky), “言” (talkative), and “虫” (cricket); therefore, his translation contains images of “bright silky warbler” and “talkative cricket,” through which new meaning may be generated.

For Pound, etymographic interpretation serves the 2-fold functions of translating classical works: to restore the lost “original” perceptions and then to bring them to interact with contemporary cultural reconstruction. However, Pound’s dependency on the etymological approach has been widely criticized because it reduces all Chinese characters to mere pictograms, while in reality the majority of Chinese characters belong to the pictophonetic category and cannot be taken as verbal pictures alone (Lan, 2005, p. 32–36). Consequently, some readers would rather see his Confucian translations as original poems. Pound’s etymographic interpretation inserts plenty of new information into the translated text, amplifying sentence length tremendously, which accounts for the rich lexicon as well as the complicated and changeable syntactic structure in his translation of *Daxue*. Nevertheless, Pound employed straightforward vocabulary in his translation to make it easy to read and capture the interest of the western public.

#### 4.2.3. Scholars since the second half of the 20th century: To excavate the profound meaning

Chan Wing-tsit (1901—1994) was a Chinese scholar and professor of Philosophy at Chatham College in America. Chan (1963) was the author of *A Source Book in Chinese Philosophy*, one of the most influential sources in Asian studies, and of hundreds of books and articles on Chinese philosophy and religion. Bloom (1995, p. 466) referred to him in his eulogy as “a living exemplar of the Chinese philosophical tradition.”

Chan (1963, p. ix) noticed that the understanding of Chinese philosophy by westerners was rather limited at that time, and stated in the preface to *A Source Book in Chinese Philosophy*: “…in order to understand the mind of China, it is absolutely necessary to understand Chinese thought, especially Neo-Confucianism, in its entire historical development.” Chan (1963, p. xi) pointed out that some Chinese terms are so complicated in meaning that there are no English equivalents for them and they therefore have to be transliterated, but he preferred to have a term translated even though the translation may not be entirely satisfactory. Chan attached great importance to the classic *Daxue*:

The importance of this little Classic is far greater than its small size would suggest. It gives the Confucian educational, moral, and political programs in a nutshell…it is the central Confucian doctrine of humanity (*jen*) in application…It is no less important from the philosophical point of view (Chan, 1963, p. 84).

Chan’s translation of *Daxue* attempts to fully maintain the original Chinese culture and the philosophical meaning of some core concepts through a combination of literal translation and transliteration. To cite a few examples, “止” was translated as “abide (*chih*)” [see Example (1)], and “格物” as “the investigation of things (*ko-wu*),” and so forth. Moreover, he also demonstrated his unique interpretation of some philosophical terms, such as the rendition of “亲民” into “loving the people” [see Example (1)], which differs from the other versions.

Andrew Plaks (1945—) is an American professor of East Asian studies at Princeton University and an authority on classical Chinese literature. Publications of Plaks include *Archetype and Allegory in the Dream of the Red Chamber (2016)*, and others. During his study of classic Chinese novels of the Ming and Qing periods, he realized the cultural and spiritual significance that *Daxue* and *Zhongyong* had for writers and readers of late-imperial China, which aroused his interest in studying and translating these two classics:

These are not massive tomes…Yet the *Ta Hsueh* (*Daxue*) and the *Chung Yung* (*Zhongyong*) have exerted an influence on the hearts and minds of men so profound and far-reaching as to bear comparison with none but the greatest monuments of the world’s major scriptural traditions (Plaks, 2003, p.xxvi-xxvii).

Plaks (2003, p. xxvi) emphasized the importance of a clear understanding of “this prime canonic source for the Confucian doctrine of moral self-cultivation,” which suggests why the word “moral” has a high-frequency use in his translation.^10^ Plaks’ translator’s note explains his translation principles of these Chinese classics: In general, he aimed to reproduce the “equivalent utterances,” that is, approximations of the semantic and syntactic values expressed in context rather than the strict matching of lexical units, so he frequently allowed himself considerable leeway in translation, by inserting additional words to convey loaded Chinese concepts (Plaks, 2003, p. xxxvi). Plaks believes that bare word-for-word rendering is inadequate, so he endeavors to draw out the multiple layers of meaning of the original work by taking advantage of both traditional glosses and modern commentaries: “I try to maximize grammatical and syntactic flexibility at the level of sentence structure as a means of finding equivalents of Chinese modes of expression” (Plaks, 2003, p.xxxvii). Plaks often inserted additional information into his translation to make the implicit meaning in the original text explicit to the target readers.

As shown in Example (1), Plaks rendered “大学之道” as “The Way of self-cultivation, at its highest level, is a 3-fold path,” adding supplementary information to clarify the meaning. Furthermore, Plaks (2003, p. xxxvii) attempted to capture the multiple “voices” of the original Chinese text, such as occasionally employing a deliberately pedantic style in English when citing ancient canonic lines. Example (5) illustrates translations by Plaks and Chan:

5. ST: 《诗》 云:“节彼南山, 维石岩岩。赫赫师尹, 民具尔瞻。”

Plaks: It is said in the *Songs*:

 ‘Lofty is that southern peak,

 its rocks piled up in towering crags.

 And towering, too, is thy authority, Marshal Yin.

 All the people look up to thee in desperate.

 supplication.’

 

Chan: The *Book of Odes* says, “Lofty is the Southern Mountain! How massive are the rocks! How majestic is the Grand Tutor Yin (of Chou)! The people all look up to you!”

In Example (5), Plaks adopts a deliberately archaic style to translate poems from the ancient *Book of Songs*, using forms like “thy” and “thee,” while Chan’s translation has a more casual style. Although Plaks and Chan are both scholarly translators and attach great importance to the original work *Daxue*, their translation approaches are markedly different, which may relate to their different socio-historical backgrounds: Plaks’ translation came out in 2003, four decades later than Chan’s translation, when the study of Chinese classics in the United States rapidly grew, with the bulk of monographs on Chinese philosophy published then. In the 1960s, when Chan’s translation was completed, few people were acquainted with the ancient Chinese thought embodied in the classics, so the key purpose of translation was to popularize the Chinese classic among general western readers, hence readability was highly important. Chan’s translation has a great number of short sentences and conjunctions to make the translated text more comprehensible to western readers, which underlies the readability level of his translation. On the other hand, Plaks’ translation targeted an audience better acquainted with Chinese culture; thus, his translation purpose was to unveil the multiple layers of the meaning in the original, to enable the target readers to appreciate the quintessential essence of the original work. Plaks’ approach is the “thick translation” put forward by Appiah, an “academic” translation that seeks with its annotations and its accompanying glosses to locate the text in a rich cultural and linguistic context (Appiah, 2000, p. 427). In addition to adding concepts/explanations to the translated text, Plaks also made use of a variety of supplementary materials, including an introduction, translation notes, chronological table, structural analysis, reader’s notes, further discussions of basic concepts, and so on, to offer a panoramic view of the Chinese classic to the target readers. Plaks had the most notes in the six versions as shown in Table 11.

**Table 11:** Comparison of the number of notes of the six TTs.

TT	Collie	Legge	Ku	Pound	Chan	Plaks
No. of Notes	24	40	5	9	44	67

Plaks made extensive use of notes to further elaborate on the difficult linguistic and philosophic issues necessary for readers to fully comprehend the profound meaning of the original. Plaks’ thick translation approach is notable for lengthy sentences and dense information load. Moreover, the usage of a difficult broad vocabulary and a significant proportion of passive sentences render his translation highly academic in style, with lower readability.

## 5. Conclusion

The translator’s voice, the translator’s discursive presence in a translated text, is always present along with the author’s voice. As a mark of the translator’s identity, the translator’s voice can be identified in the body of the translated text as well as the paratexts of translation. To start with, this study took a corpus-based approach to investigate the translator’s voice in six English translations of the Chinese classic *Daxue* by tracing the linguistic cues in each version. The findings show the variety of styles through the different lexical, syntactic, and textual features. Next, this study investigated the paratexts of translation and elaborated on the translators’ cultural identities and translation motives within their particular socio-historical contexts by analyzing representative examples. The combination of qualitative and quantitative analyses brought to light the translators’ voices in the different versions of *Daxue*. In summary, the 19th-century missionaries tended to maintain the linguistic and stylistic features of the original for the most part since their main purpose was to learn Chinese to facilitate missionary work. However, they took advantage of prefaces, introductions, lengthy footnotes, and so on, to ventilate their opinions. In other words, their voices as translators were most strongly felt in the paratexts of translation. Although both Collie and Legge sought to evangelize Christianity, Legge placed more value on having a genuine understanding of the Chinese culture; thus in his translation, faithfulness and readability were more accentuated. In the turbulent first half of the 20th century, translators’ voices were more overtly present with an obtrusive intervention in the body of translated texts, adapting the meaning and style of the original to produce a more creative translation as in Ku’s domestication approach and Pound’s etymographic interpretation. Their translations took a more personal style and had a more powerful emotional pull. Since the second half of the 20th century, translators have demonstrated a propensity to elicit the nuanced and intricate meaning of the original, in an effort to recapture the genuine essence of the classic. Especially in the 21st century, “thick translation” is adopted which adds information to the translated text and makes full use of a variety of paratextual materials to satisfy the demand of an increasingly knowledgeable readership. Consequently, profound comprehension of the original allows the translator’s voice to break through the surface of the original declaring its presence both in the body of the translated text and in the paratexts of translation as well more overtly.

To conclude, a better and more thorough understanding of the English translations of *Daxue* can be gained by examining the diverse textual cues in the translated texts and the paratexts of translation, as well as the socio-historical contexts associated with them. A comparative analysis of parallel corpora helped identify the linguistic similarities and differences in the six translated texts, revealing the translators’ voices. However, it should be noted that the features identified in the current study are limited to the translation of this particular text rather than those of the translators under discussion in general, or to the analysis of other works. Hopefully, more research will be conducted in this area based on corpus analysis in the future. The current study has demonstrated how corpus-based statistical analysis serves as a practical entry point for the study of the translation of Chinese classics and exploring the translator’s voice.

## Data availability statement

The original contributions presented in the study are included in the article/supplementary material, further inquiries can be directed to the corresponding author.

## Author contributions

The author confirms being the sole contributor of this work and has approved it for publication.

## Conflict of interest

The author declares that the research was conducted in the absence of any commercial or financial relationships that could be construed as a potential conflict of interest.

## Publisher’s note

All claims expressed in this article are solely those of the authors and do not necessarily represent those of their affiliated organizations, or those of the publisher, the editors and the reviewers. Any product that may be evaluated in this article, or claim that may be made by its manufacturer, is not guaranteed or endorsed by the publisher.

## Footnotes

^1^The reprinted editions which are nearly identical to their first editions are not listed. The translation of *Daxue* by Ku Hungming was completed in 1915 but published later. The two English versions of *Daxue* by Ezra Pound are remarkably different from each other, so both are presented in the table.

^2^This paper adopts the term “the translation style” to refer to the distinctive features that differentiate one translated text from other translated texts based on the same source text and into the same target language by multiple translators.

^3^The Chinese original of *Daxue* is taken from *Sishu Zhangju Jizhu* (*Collected Annotations of The Four Books*) compiled by Zhu Xi, published by Zhonghua Book Company in 2011, excluding Zhu Xi’s annotations.

^4^The translation by Ku Hungming in 1915-*Higher Education* is included in *The Discourses and Sayings of Confucius* (*The Analects*), *Higher Education*, *The Conduct of Life*, *or The Universal Order of Confucius: A Bilingual Edition* published by Tianjin Social Sciences Academy Press in 2016.

^5^ http://ucrel.lancs.ac.uk/claws/trial.html

^6^ https://datayze.com/readability-analyzer

^7^The Flesch score uses the number of syllables and sentence lengths to determine the reading ease of the sample. A Flesch score of 60 is taken to be plain English. A score in the range of 60–70 corresponds to the 8th/9th grade English level. A score between 50 and 60 corresponds to the 10th/12th grade level. Below 30 is college graduate level.

^8^According to Pellatt (2013, p. 1), paratexts refer to “any material additional to, appended to or external to the core text which has functions of explaining, defining, instructing, or supporting, adding background information, or the relevant opinions and attitudes of scholars, translators and reviewers.”

^9^According to the frequency list generated by the Wordsmith Concord Program, Ku’s translation contains 16 instances of the word “moral,” while the cluster “moral qualities/quality” appears eight times.

^10^According to the frequency list generated by the Wordsmith Concord Program, Plaks’ translation contains 26 instances of the word “moral,” while the cluster “moral character” appears six times.

## References

[ref1] Alexander M. (1979). The Poetic Achievement of Ezra Pound. London and Boston: Faber & Faber.

[ref2] Appiah K. A. (2000). “Thick translation,” in The Translation Studies Reader. ed. Venuti L. (London and New York: Routledge), 417–429.

[ref3] Baker M. (1995). Corpora in translation studies: An overview and some suggestions for future research. Target. Int. J. Transl. Stud. 7, 224–243.

[ref4] Baker M. (1996). “Corpus-based translation studies: the challenges that lie ahead,” in Terminology, LSP and Translation: Studies in Language Engineering, in Honour of Juan C. Sager. ed. Somers H. (Amsterdam: John Benjamins), 175–186.

[ref5] Baker M. (2000). Toward a methodology for investigating the style of a literary translator. Targets 12, 241–266. doi: 10.1075/target.12.2.04bak, PMID: 27409075

[ref6] Bloom I. (1995). Remembering wing-tsit Chan by Bloom. Philos. East West 45, 466–471.

[ref7] Chai C. (1965). The Sacred Book of Confucius, and Other Confucian Classics. New York: University Books.

[ref8] Chan W. T. (1963). A Source Book in Chinese Philosophy. Princeton: Princeton University Press.

[ref9] Chatman S. (1978). Story and Discourse: Narrative Structure in Fiction and Film. Ithaca, NY: Cornell University Press.

[ref10] Cheadle M. P. (1997). Ezra Pound’s Confucian Translations. Ann Arbor: The University of Michigan Press.

[ref11] Chen Q. (2020). Comments on and analysis of the translations of the *Daxue* by English Protestant missionaries in the 19th century. East J. Transl. 8, 4–11.

[ref12] Collie D. (1828). The Chinese Classical Work Commonly Called the Four Books. London: The Mission Press.

[ref13] De Bary W. T. Bloom I. (1999). Sources of Chinese Tradition: From Earliest Times to 1600 (2nd Edn.). New York: Columbia University Press.

[ref14] Eno R. (2016). The great learning and the doctrine of the mean: translation, commentary and notes. Available at: http://hdl.handle.net/2022/23424 (Accessed October 10, 2022).

[ref15] Fan M. (2017). A corpus-based study of the translation of the cultural high-frequency words in the five versions of Lunyu. For. Lang. Educ. 38, 80–83. doi: 10.16362/j.cnki.cn61-1023/h.2017.06.015

[ref16] Fang M. Z. (2011). Comparison of the translations of the Daxue. J. Hubei Corresp. Univ. 24, 133–134. doi: 10.3969/j.issn.1671-5918.2011.10-066

[ref17] Ge H. W. (2019). A study of the translator’s style of Shangshu based on corpus. Yangzhou University.

[ref18] Hall S. (1990). “Cultural identity and diaspora,” in Identity Community, Culture, Difference. ed. Rutherford J. (London: Lawrence & Wishart), 222–237.

[ref19] He Z. K. (1996). The Great Learning, the Doctrine of the Mean. Beijing: Sinolingua Press.

[ref20] Hermans T. (1996). The translator’s voice in translated narrative. Target: international journal of. Transl. Stud. 8, 23–48. doi: 10.1075/target.8.1.03her

[ref21] Hermans T. (2014). Positioning translators: voices, views and values in translation. Lang. Lit. 23, 285–301. doi: 10.1177/0963947014536508

[ref22] Hou J. (2019). E. R. Hughes and his translation of the great learning and the doctrine of mean. Int. Sinol. 4, 45–52. doi: 10.19326/j.cnki.2095-9257.2019.04.006

[ref23] Hu K. B. Xie L. X. (2017). Towards a corpus-based study of translator’s style. Chin. Transl. J. 2, 12–18.

[ref24] Huang L. B. (2018). Reflections on corpus-based studies of the translator’s style. For. Lang. Educ. 39, 77–81. doi: 10.16362/j.cnki.cn61-1023/h.2018.01.016

[ref25] Hughes E. R. (1942). The Great Learning and the Mean-In-Action. New York: E. P. Dutton and Company.

[ref26] Johnston I. Wang P. (2012). Daxue and Zhongyong (Bilingual Edition). Hongkong: The Chinese University Press.

[ref27] Ku H. M. (1920). The Conduct of Life or the Universal Order of Confucius. London: John Murray.

[ref28] Ku H. M. (2016). The Discourses and Sayings of Confucius (the analects), Higher Education, the Conduct of Life, or the Universal Order of Confucius: A Bilingual Edition. Tianjin: Tianjin Social Sciences Academy Press.

[ref29] Lan F. (2005). Ezra Pound and Confucianism: Remaking Humanism in the Face of Modernity. Toronto: University of Toronto Press Incorporated.

[ref30] Lefevere L. (1977). Translating Literature: The German Tradition From Luther to Rosenzweig. Assen: Van Gorcum.

[ref31] Legge J. (1861). The Chinese Classics: With a Translation, Critical and Exegetical Notes, Prolegomena 1. Hongkong & London: Trubner & co.

[ref32] Lin Y. T. (1938). Wisdom of Confucius. New York: Random House.

[ref33] Lu X. Y. (2013). A study of Legge’s translation of the great learning from the perspective of translation as adaptation and selection. Master’s thesis. Shanghai: Donghua University.

[ref34] Marshman J. (1814). Elements of Chinese Grammar. Serampore: The Mission Press.

[ref35] Morrison R. (1812). Horae Sinicae: Translations From the Popular Literature of the Chinese (Trans.). London: Black and Parry.

[ref36] Muller A. C. (1992). Great learning. Available at: http://www.acmuller.net/con-dao/index.html

[ref37] Munday J. (2008). Style and Ideology in Translation: Latin American Writing in English. New York: Routledge.

[ref38] Munday J. (2009). The creative voice of the translator of Latin American literature. Roman. Stud. 27, 246–258. doi: 10.1179/026399009X12523296128795

[ref39] O’Sullivan O. (2005). Comparative Children’s Literature (A. Bell, Trans.). London & New York: Routledge.

[ref40] Olohan M. (2004). Introducing Corpora in Translation Studies. London & New York: Routledge.

[ref41] Pellatt V. (2013). Text, Extratext, Metatext and Paratext in Translation. Newcastle upon Tyne: Cambridge Scholars Publishing.

[ref42] Plaks A. (2003). Ta Hsuch and Chung Yung: The Highest Order of Cultivation and on the Practice of the Mean. London: Penguin Books Ltd.

[ref44] Pound E. (1928). Ta Hio, The Great Learning of Confucius. Washington: University of Washington Book Store.

[ref45] Pound E. (1947). The Great Digest & Unwobbling Pivot (Trans.). New Jersey: Blue Ridge Mountain Press.

[ref46] Taivalkoski-Shilov K. (2015). Friday in Finnish: a character’s and (re)translators’ voices in six Finnish retranslations of Daniel Defoe’s Robinson Crusoe. Targets 27, 58–74. doi: 10.1075/target.27.1.03tai

[ref47] Venuti L. (1995). The Translator’s Invisibility: A History of Translation. London and New York: Routledge.

[ref48] Wallwork A. (2016). English for Academic Research: Grammar, Usage and Style. New York: Springer.

[ref49] Wang H. (2005). The etymographic interpretation and Pound’s translation of the Daxue. Transl. Q. 38, 62–82.

[ref50] Wang H. Ye L. M. (2008). The politics of “literal translation”: comments on the translation of the *Daxue* by Robert Morrison. J. Guangdong Univ. For. Stud. 3, 50–62.

[ref51] Wang H. Ye L. M. (2009). A comparison of Robert Morrison’s and Joshua Marshman’s translations of the Daxue. J. Chin. Stud. 49, 413–426.

[ref52] Wu G. Z. (2018). Three Confucian Classics With Relevant Paraphrases. Beijing: Beijing Press.

[ref53] Xu X. (2020). A study on the translation style of the English versions of the *Daxue* based on text data mining. East J. Transl. 5, 36–42.

[ref54] Zhao Y. (2015). A corpus-based study on the translation style of the two English versions of *Daodejing*. Chin. Transl. J. 4, 110–113.

[ref55] Zhu C. G. (2005). Ezra Pound’s Confucianism. Philos. Lit. 29, 57–72. doi: 10.1353/phl.2005.0016

[ref56] Zhu X. (2011). Sishu Zhangju Jizhu(Collected Annotations of the Four Books). Beijing: Zhonghua Book Company.

